# Supplementary material for: Excess ribosomal protein production unbalances translation in a model of Fragile X Syndrome
Source: Nat Commun. 2022 Jun 10;13:3236. doi: 10.1038/s41467-022-30979-0 (PMC9187743; doi:10.1038/s41467-022-30979-0)
Supplement: Supplementary file 2 — Description of Additional Supplementary Files [file 41467_2022_30979_MOESM2_ESM.pdf]

Description of Additional Supplementary Files

Supplementary Data 1. Contains raw data from the *Fmr1*$^{-/y}$ proteomics analysis

Supplementary Data 2. Contains GSEA analyses of TRAP-seq and proteomics datasets

Supplementary Data 3. Contains raw data from *Fmr1*$^{-/y}$ SNAP-TRAP-seq

Supplementary Data 4. Contains raw data from DHPG TRAP-seq

Supplementary Data 5. Contains GSEA analyses of DHPG TRAP-seq

Supplementary Data 6. Lists targets upregulated in both WT DHPG TRAP-seq and in *Fmr1*$^{-/y}$ CA1-TRAP-seq

Supplementary Data 7. Lists RPs upregulated in both WT DHPG TRAP-seq and in *Fmr1*$^{-/y}$ CA1-TRAP-seq

Supplementary Data 8. Contains GSEA analyses of transcript lengths in TRAP-seq

Supplementary Data 9. Lists targets downregulated in both *Fmr1*$^{-/y}$ proteomics and *Fmr1*$^{-/y}$ SNAP-TRAP-seq datasets

Supplementary Data 10. Lists targets downregulated in both *Fmr1*$^{-/y}$ proteomics and *Fmr1*$^{-/y}$ CA1-TRAP-seq datasets

Supplementary Data 11. Contains raw data from DHPG transcriptome

Supplementary Data 12. Lists targets downregulated in both WT DHPG TRAP-seq and in *Fmr1*$^{-/y}$ CA1-TRAP-seq

Supplementary Data 13. Contains raw data from CX-5461 DHPG TRAP-seq
