# Supplementary material for: Excess ribosomal protein production unbalances translation in a model of Fragile X Syndrome
Source: Nat Commun. 2022 Jun 10;13:3236. doi: 10.1038/s41467-022-30979-0 (PMC9187743; doi:10.1038/s41467-022-30979-0)
Supplement: Supplementary file 16 — Reporting Summary [file 41467_2022_30979_MOESM16_ESM.pdf]

nature research

Corresponding author(s): Emily K Osterweil

Last updated by author(s): 4/27/2022

Reporting Summary

Nature Research wishes to improve the reproducibility of the work that we publish. This form provides structure for consistency and transparency in reporting. For further information on Nature Research policies, see Authors & Referees and the Editorial Policy Checklist .

Statistics

For all statistical analyses, confirm that the following items are present in the figure legend, table legend, main text, or Methods section.

n/a	Confirmed	
☐	☒	The exact sample size (*n*) for each experimental group/condition, given as a discrete number and unit of measurement
☐	☒	A statement on whether measurements were taken from distinct samples or whether the same sample was measured repeatedly
☐	☒	The statistical test(s) used AND whether they are one- or two-sided<br>*Only common tests should be described solely by name; describe more complex techniques in the Methods section.*
☐	☒	A description of all covariates tested
☐	☒	A description of any assumptions or corrections, such as tests of normality and adjustment for multiple comparisons
☐	☒	A full description of the statistical parameters including central tendency (e.g. means) or other basic estimates (e.g. regression coefficient) AND variation (e.g. standard deviation) or associated estimates of uncertainty (e.g. confidence intervals)
☐	☒	For null hypothesis testing, the test statistic (e.g. *F*, *t*, *r*) with confidence intervals, effect sizes, degrees of freedom and *P* value noted<br>*Give P values as exact values whenever suitable.*
☒	☐	For Bayesian analysis, information on the choice of priors and Markov chain Monte Carlo settings
☒	☐	For hierarchical and complex designs, identification of the appropriate level for tests and full reporting of outcomes
☐	☒	Estimates of effect sizes (e.g. Cohen's *d*, Pearson's *r*), indicating how they were calculated

Our web collection on statistics for biologists contains articles on many of the points above.

Software and code

Policy information about availability of computer code

Data collection	Proteomics data were collected using Spectronaut 13.7
Data analysis	Proteomics data were analysed using Limma R package 3.40.6 (Bioconductor). FDRs were computed with fdrtool R package version 1.2.16. Individual gene plots were created with DEP R package version 1.6.1. For RNA-seq, reads were mapped to the Mus musculus primary assembly (Ensembl release v88) using STAR 2.4.0i. Reads that were uniquely aligned to annotated genes were counted with featureCounts 1.4.6-p2. Differential expression analyses were performed using DESeq2_1.18.1 (Bioconductor). All GSEA and GSA were performed with Piano package version 1.18.1 (Bioconductor). Custom script used to calculate 5'UTR length is available at https://github.com/sidbdri/empirical_utrs. Imaging analyses were performed using IMARIS 9.1.7 & 9.2.0. Desitometry of immunoblots was performed using ImageStudio Lite v5.0 and FIJI v1.51.

For manuscripts utilizing custom algorithms or software that are central to the research but not yet described in published literature, software must be made available to editors/reviewers. We strongly encourage code deposition in a community repository (e.g. GitHub). See the Nature Research guidelines for submitting code & software for further information.

Data

Policy information about availability of data

All manuscripts must include a data availability statement. This statement should provide the following information, where applicable:

- Accession codes, unique identifiers, or web links for publicly available datasets
- A list of figures that have associated raw data
- A description of any restrictions on data availability

The mass spectrometry proteomics data generated in this study have been deposited to the ProteomeXchange Consortium via the PRIDE, partner repository with the dataset identifier PXD031932 ((Fig. 1, Fig. 4, Supplementary Fig. 1, Supplementary Fig. 3). The RNAseq data generated in this study has been deposited in GEO with the dataset identifiers: GSE199328 (https://www.ncbi.nlm.nih.gov/geo/query/acc.cgi?acc=GSE199328) for SNAP TRAP dataset (Fig. 1, Fig. 4, Supplementary Fig. 1, Supplementary Fig.3), GSE201239 (https://www.ncbi.nlm.nih.gov/geo/query/acc.cgi?acc=GSE201239) for DHPG TRAP (Fig. 2, Fig. 5, Supplementary Fig. 2,

Supplementary Fig. 4) and GSE200919 (https://www.ncbi.nlm.nih.gov/geo/query/acc.cgi?acc=GSE200919) for the CX DHPG dataset (Fig. 6, Supplementary Fig. 5). Additional data generated in this study is provided in the Supplementary Information and Source Data file. All renewable reagents and protocols will be available upon reasonable request.

Field-specific reporting

Please select the one below that is the best fit for your research. If you are not sure, read the appropriate sections before making your selection.

[x] Life sciences [ ] Behavioural & social sciences [ ] Ecological, evolutionary & environmental sciences

For a reference copy of the document with all sections, see nature.com/documents/nr-reporting-summary-flat.pdf

Life sciences study design

All studies must disclose on these points even when the disclosure is negative.

Sample size	Sample sizes for were chosen to minimize sample-to-sample variability as determined in previous studies (Thomson et al., 2017; Osterweil et al., 2013).
Data exclusions	For molecular biology and biochemistry experiments, outlier analyses were performed however no data points were ultimately excluded. For LTD experiments unstable recordings (baseline drift +/- 5%) were removed from analysis prior to unblinding. For the CX+DHPG TRAP-seq dataset, one sample was excluded based on poor quality read mapping.
Replication	Experimental samples were taken from multiple littermate pairs on several different experimental days. All results shown are averages of at least 3 independent experiments. With the exception of LTD recordings, each experiment contained yoked controls for genotype and treatment. The variability of effect sizes between yoked samples can be seen in the individual data points in each plot.
Randomization	Littermate male mice were assigned to groups randomly and interleaving performed wherever possible.
Blinding	All experiments and analyses were performed blind to genotype and treatment.

Reporting for specific materials, systems and methods

We require information from authors about some types of materials, experimental systems and methods used in many studies. Here, indicate whether each material, system or method listed is relevant to your study. If you are not sure if a list item applies to your research, read the appropriate section before selecting a response.

Materials & experimental systems

n/a	Involved in the study	
[ ]	[x]	Antibodies
[x]	[ ]	Eukaryotic cell lines
[x]	[ ]	Palaeontology
[ ]	[x]	Animals and other organisms
[x]	[ ]	Human research participants
[x]	[ ]	Clinical data

Methods

n/a	Involved in the study	
[x]	[ ]	ChIP-seq
[x]	[ ]	Flow cytometry
[x]	[ ]	MRI-based neuroimaging

Antibodies

Antibodies used	The following antibodies were diluted according to the manufacturer's specifications listed for immunoblotting, immunostaining or flow cytometry: Rpl10a (Abcam 174318), Rps25 (Thermo scientific PA5-56865), Rps4x (Proteintech 14799-1-APtwo), Donkey anti-rabbit Alexa Fluor 594 conjugated secondary antibody (Thermo Fisher R37119), anti-Y10b (Abcam, ab171119), anti-NeuN (Merck, ABN78), anti-Fibrillarin (Abcam, ab5821), anti-NeuN (Millipore, MAB377), Alexa Fluor 488 conjugated anti-NeuN (Abcam, ab190195), Donkey anti-rabbit and donkey anti-mouse HRP-linked secondary antibodies (Cell Signaling Technology 7074, 7076). The dilutions used for each experiment were determined by the range provided with the antibody specification sheet.
Validation	Validation statements for all antibodies used were supplied by the manufacturer for the species and specification. Information on this validation can be found on the manufacturer's website for each antibody listed.

Animals and other organisms

Policy information about studies involving animals; ARRIVE guidelines recommended for reporting animal research

Laboratory animals	Fmr1-/y (JAX 003025), Snap25-EGFP/Rpl10a (JAX 030273), and CA1-TRAP mice (JAX GM391-TRAP) were bred on a C57BL/6J background. Fmr1-/y and WT littermates were bred using Fmr1+/- females and JAX C57BL/6J males. Fmr1-/y-TRAP and WT-TRAP littermates were bred using Fmr1+/- females and TRAP homozygous males. All experiments were carried out using male

	littermate mice aged P25-32 with the experimenter blind to genotype. Mice were group housed (6 maximum) in conventional non-environmentally enriched cages with unrestricted food and water access and a 12h light-dark cycle. Room temperature was maintained at 21 ± 2°C with ambient humidity.
Wild animals	No wild animals were used in this study.
Field-collected samples	No field collected samples were used in this study.
Ethics oversight	All procedures were performed in accordance with ARRIVE guidelines and the UK Animal Welfare Act 2006, and were approved by the Animal Welfare and Ethical Review Body at the University of Edinburgh.

Note that full information on the approval of the study protocol must also be provided in the manuscript.
